# Turkish Translation, Cross-Cultural Adaptation, Validity, and Reliability of the Physical Activity and Social Support Scale (PASSS) in Physically Active Healthy Adults

**DOI:** 10.3390/healthcare13111343

**Published:** 2025-06-05

**Authors:** Yıldız Analay Akbaba, Büşra Aksan Sadıkoğlu, Kübra Nur Menengiç, Meltem Besim Atakan, Doğukan Tongar, Gulfidan Tokgoz, Alper Ayas, Sahra Şirvan Tongar, Tuğba Akgüller Eker

**Affiliations:** 1 Division of Physiotherapy and Rehabilitation, Faculty of Health Science, Istanbul University-Cerrahpasa, Istanbul 34320, Türkiye; yildizanalay@iuc.edu.tr; 2 Institute of Graduate Studies, Department of Physical Therapy and Rehabilitation, Istanbul University-Cerrahpasa, Istanbul 34320, Türkiye; kmenengic@gmail.com (K.N.M.); meltembesim@gmail.com (M.B.A.); dogukan.tongar@yeditepe.edu.tr (D.T.); gulfidantokgoz@gmail.com (G.T.); alperayas290@gmail.com (A.A.); sahrasirvan.can@ogr.iuc.edu.tr (S.Ş.T.); t.akguller09@gmail.com (T.A.E.); 3 Division of Physical Therapy and Rehabilitation, Faculty of Health Sciences, Istanbul Kent University, Istanbul 34433, Türkiye; 4 Health Institutes of Türkiye, Istanbul 34718, Türkiye; 5 Division of Physical Therapy and Rehabilitation, Faculty of Health Sciences, Yeditepe University, Istanbul 34755, Türkiye; 6 Division of Therapy and Rehabilitation, Bigadiç Vocational School, Balikesir University, Balikesir 10440, Türkiye

**Keywords:** emotional support, physical activity, healthy adult, social support, Turkish validation

## Abstract

**Background/Objectives:** The Physical Activity and Social Support Scale (PASSS) is used to evaluate the physical activity and social support in a multidimensional way, but it has not yet been translated or culturally adapted for Turkish-speaking individuals. The aim of this study is to evaluate the validity and reliability of the Turkish version of the PASSS, which evaluates social support for physically active, healthy young adults. **Methods:** Two hundred and two individuals (98 females, 104 males; mean ± SD age, 26.5 ± 6.1 years; BMI, 23.3 ± 3.2 kg/m^2^) participated in the study. The PASSS was translated into Turkish using the Beaton guidelines. Multidimensional Scale of Perceived Social Support (MSPSS), International Physical Activity Questionnaire-Short Form (IPAQ-SF), and Short Form-12 Health Survey (SF-12) were used for construct, convergent, and divergent validity. **Results:** The PASSS demonstrated good internal consistency (Cronbach’s α = 0.84) and excellent reliability (ICC = 0.90; 95% CI = 0.86–0.93). The PASSS showed good correlation with the MSPSS (r = 0.378, *p* = 0.001), fair correlation with the IPAQ-SF (r = 0.271, *p* = 0.001), and poor correlation with the SF-12 physical component score (PCS-12) (r = 0.15, *p* = 0.03); it was not correlated with the SF-12 mental component score (MCS-12) (r = 0.102 *p* = 0.15). We observed no ceiling and floor effects. **Conclusions:** The results show that the PASSS Turkish version is reliable and valid and can be utilized for physically active, healthy young Turkish adults.

## 1. Introduction

Physical activity is described as any bodily movement produced by skeletal muscles that requires a certain amount of energy expenditure. The fact that the current physical activity recommendations are not fulfilled or not at a sufficient level is expressed as physical inactivity. The rate of physical inactivity in adults varies between countries and can reach up to 80% for certain countries [1]. WHO aims to reduce physical inactivity in adults and adolescents by 15% through the global action plan published in 2019 [2]. Biological and demographic factors and psychosocial factors are among the factors that may cause sedentary behavior. Many behavioral change and health behavior adoption theories emphasize the importance of social support and psychological factors for maintaining a physically active lifestyle [3,4]. In many studies involving a psychosocial variable, social support was found to be correlated with physical activity levels [5].

Social support has been defined as any behavior that helps a person achieve a desired goal [3]. This support can be provided from various sources (family, friends, etc.) in the social environment of the individual. Social support can help individuals mitigate or alleviate the negative experiences of stressful situations by improving their coping skills [4]. Therefore, social support is expected to have a significant impact on physical activity behaviors. Although there are positive results of investigating the relationship between social support and physical activity, studies lack evaluation for functional forms. Functional social support describes the perceived support available to adults when coping with chronic and acute stress [6]. Functional social support is basically expressed in 5 different ways: emotional, companionship, instrumental, informational, and validation support [6,7,8,9,10,11]. Although these forms of social support are widely used in other health fields, it has been seen that only emotional, companionship, and instrumental support are included in the studies on physical activity [9,10]. Therefore, the current literature does not provide sufficient information or validation support for physical activity.

In the current literature, five valid scales of social support for physical activity are used: (a) “Social Support and Exercise Questionnaire”, (b) “Social Provisions Scale”, (c) “Child and Adolescent Social Support Scale”, (d) “Adolescent Social Support Scale for International Physical Activity (ASAFA)”, and (e) “Physical Activity and Social Support Scale—PASSS” [12,13,14,15,16]. When the first three scales are examined, it is seen that these scales limit the types of social support to family or friends and are inadequate in addressing multiple forms of social support. Moreover, these scales do not provide a comprehensive assessment of functional social support.

The PASSS, developed by Golaszewski and Bartholomew (2019), is a scale designed to assess the level of social support for physical activity [16]. This scale allows for the comprehensive assessment of the effect of social support on physical activity across five categories; these include companionship, emotional, instrumental, informational, and validation [16]. In addition, instead of limiting the support sources to only family or friends, it takes into account multiple sources of support, such as live or online groups, internet blogs, and online searches.

PASSS has recently been validated across diverse populations and countries, demonstrating strong psychometric properties. For instance, Zou et al. (2023) conducted a comprehensive psychometric evaluation of the Chinese version of PASSS (PASSS-C) in a large sample of adults aged 30–45 years [17]. Their findings confirmed the structural validity and internal consistency of the scale, supporting its utility for assessing the social support related to physical activity in this population [17]. In addition, PASSS has been used in various contexts—including group exercise participants, Attention Deficit Hyperactivity Disorder (ADHD), underserved minorities, nursing students, and cancer survivors—to assess different forms and sources of social support related to physical activity [18,19,20,21]. Golaszewski et al. (2022) report that belonging to an exercise group is associated with forms of social support that strengthen exercise identity [22]. It is stated that belonging to an exercise group provides all five types of social support for females, while for males, it provides emotional, validation, informational, and companionship support, but not instrumental [16]. Studies using PASSS with children with ADHD and underrepresented minorities have emphasized that social support is a crucial factor in promoting participation in physical activity [18,19]. Additionally, a study with nursing students highlighted that emotional and instrumental social support were associated with the reduction of barriers to physical activity participation [20]. Furthermore, coach-led group exercise training was found to lead to improvements in the companionship sub-dimension of PASSS among cancer survivors. It has been stated that the social interaction within the group encouraged participants to engage more actively and attend the program more consistently, thereby leading to greater benefits in physical activity [21]. These studies underscore the importance of measuring all five functional support types and considering social support beyond close social ties. Understanding the subdimensions of social support in physical activity is crucial, as it allows for a deeper insight into the specific impact of each support type on participation. Given the growing global recognition of the role of multidimensional social support in promoting physical activity, there is a clear need for culturally adapted and psychometrically sound tools such as PASSS to enable accurate assessments, guide interventions, and support comparative research across populations.

When the existing physical activity and social support scales translated into Turkish are examined (see Supplementary Materials), it is seen that the number of studies is limited. Recently, Turkish adaptation and validation studies have been published for the “Child and Adolescent Social Support Scale” [23] and “Adolescent Social Support Scale for International Physical Activity (ASAFA)” [24]. However, these scales are specific to certain age groups and do not comprehensively assess individuals using five different forms of social support, which limits their applicability. In addition, these scales only consider parents or peers as sources of support and neglect other potential sources of social support. Although the topic of the effect of social support on physical activity has become an increasing trend in recent years, studies in Turkish have been limited to specific age groups due to the limitations of scales with Turkish validity [25,26]. To expand research to a broader population and gain a deeper understanding of the role of social support in physical activity, it is crucial to adapt and validate the PASSS in Turkish.

Considering the gaps in the existing Turkish literature, adapting the PASSS to Turkish and validating it for physically active, healthy adults in Turkish society will allow for a comprehensive assessment of the impact of five different types of social support on physical activity. This will facilitate the implementation of targeted interventions to increase the adherence to physical activity.

The aim of this study is to determine the reliability, validity, and cultural adaptation of the Turkish version of the PASSS, which measures all types of social support and its relationship with physical activity, in young adult individuals.

## 2. Materials and Methods

### 2.1. Study Design

The research was carried out between August 2022 and November 2022 with students and staff from various universities in Türkiye and was approved by the Istanbul University-Cerrahpasa Clinical Research Ethics Committee (Approval No: 2022/76). In order to investigate the validity and reliability of the scale, the number of items in the tested scale was taken into account in determining the number of samples to be included in the study (20 items in total). The sample size for validity was determined by multiplying the number of items [27] by 10, resulting in a minimum required sample size of 200 participants. A sample size of at least 50 participants is recommended as sufficient for the reliability analysis [27]. Additionally, permission to use the original scale was secured by contacting its developers via email. Inclusion criteria were as follows: participants must be between the ages of 18 and 35, participate in any form of physical activity at least twice a week, and be able to understand and speak Turkish. Individuals who have severe physical activity limitations for any reason and are diagnosed with a disease causing physical and cognitive impairment were excluded.

The subjects who participated in this study were informed about the purpose, duration, content, and possible situations of the study. Their verbal consent was obtained and recorded via “Google Forms”. Assessments were performed via telephone interview. In addition, a questionnaire prepared for this study was used to collect sociodemographic characteristics and information about physical activity habits. To assess the test–retest reliability of the Turkish version of the PASSS, participants were requested to complete the scale a second time 7–10 days after the first completion of the scale.

### 2.2. Translation and Cultural Adaptation

In this study, the PASSS was translated into Turkish with the support of its developer [NG]. The translation and cultural adaptation process followed the methodology outlined by Beaton [28].

Initially, the original scale was independently translated into Turkish by two native speakers—one physiotherapist and one professional translator. In the next phase, a bilingual expert reviewed these translations to identify and resolve any conceptual inconsistencies. Following this, the finalized Turkish translation was back-translated into English by two native English speakers fluent in Turkish. A consensus version was then developed based on these back-translations and compared with the original PASSS by an expert committee comprising methodologists, linguists, and translators to ensure equivalence. The committee reviewed the semantic, idiomatic, experiential, and conceptual equivalence of the translated version, comparing it with the original version. They presented their findings to the authors in a written report. After confirming its conceptual and linguistic consistency, the Turkish version of PASSS was finalized and approved. In order to evaluate the clarity and understandability of the Turkish version of the PASSS, a pilot test was conducted with 10 people who met the eligibility criteria. When the person filled out the questionnaire, the physiotherapist met with the person to inquire whether they had difficulty in understanding the questions. After the participant completed the questionnaire, the physiotherapist inquired whether they experienced any difficulties in understanding the questions and documented any items that were unclear [28].

### 2.3. Outcome Measures

Demographic Form: A form specially prepared by the researchers was used, in which the demographic information of the participants, such as name, surname, gender, age, body mass index (BMI), and level of education, was collected. Additionally, the type of physical activity the participants engaged in was recorded.

Physical Activity and Social Support Scale: The scale measures the relationship between social support and physical activity. We used Turkish version of scale that we translated and adapted into Turkish. The questionnaire consists of 20 questions [16].

Multidimensional Scale of Perceived Social Support: The questionnaire measures the adequacy of social support and has 12 items for assessment. The 3 subgroups include items related to the social support of friends, family, and a special individual. While the sum of the 4-item scores for each group creates the subscore, the sum of all the subscores creates the total score [29].

International Physical Activity Questionnaire-Short Form (IPAQ-SF): It was developed by Booth in 1996. The scale is used to measure the health and physical activity levels of society. A short form of the scale has been developed that provides information about walking, sedentary, moderate, and vigorous activity time. It combines the estimation of the metabolic equivalent (MET) energy consumption of the task for each category with the reported weekly duration of participation in those activities [30].

Short Form-12 Health Survey (SF-12): Adapted from Short Form-36 Health Survey (SF-36), SF-12 includes 8 sub-dimensions including 2 items for physical functionality. These include 2 items for physical role, 1 item for body pain, 1 item for general health, 1 item for social functionality, 1 item for energy, 2 items for emotional role, and 2 items for mental health—a total of 12 items. The physical (PCS-12) and mental component score (PCS-12) are calculated separately [31].

### 2.4. Statistical Analysis

SPSS (Statistical Package for Social Sciences) (SPSS 21.0) statistical program was used in the statistical analysis of the data. Descriptive statistics were computed for each of the variables. Frequency values, percentages, and central tendency measurements were calculated for distribution measurements, and categorical variables were calculated for continuous variables. The Shapiro–Wilk test was used to evaluate whether the data were suitable for normal distribution. For the measure of the internal consistency of the PASSS, the Cronbach’s α coefficient was calculated. For the Cronbach’s α coefficient, which shows the homogeneity between the items in the scale, <0.50 is considered “unacceptable”, 0.50–0.59 “weak”, 0.60–0.69 “acceptable”, 0.70–0.89 “good”, and ≥0.90 “excellent” [32]. Reliability was evaluated for the Intraclass Correlation Coefficient (ICC) as follows: ≤0.40 is “poor”, 0.41–0.60 is “moderate”, 0.61–0.80 is “good”, and ≥0.81 is “excellent” [33]. For the construct validity, correlations between PASSS and MSPSS and IPAQ-SF were assessed. The relationship between PASSS and PCS-12 for convergent validity and between PASSS and MCS-12 for divergent validity was assessed. Correlation values of 0.4 or higher were deemed acceptable, with the following classifications: 0–0.2 as “poor”, 0.21–0.4 as “fair”, 0.41–0.6 as “good”, 0.61–0.8 as “very good”, and r ≥ 0.81–1.0 as “excellent” [27]. The Mann–Whitney U test was used to analyze the differences between groups.

The ceiling and floor effects of the first and last evaluations of the PASSS were evaluated by calculating the ratio of the participants who received the maximum or minimum values from the scale to all the participants. Scores between 90% and 100% were considered the maximum, and scores between 0% and 10% were considered the minimum. Ceiling and floor effects were taken into account if more than 30% of scores were within the limits of the scale [34]. For all tests, statistical significance value was set at *p* < 0.05.

## 3. Results

### 3.1. Translation Process of the PASSS

According to the interpretations of the translators, there were some matching English words with Turkish words, like the word ‘reassurance’ in item 1, ‘emotional support/assurance (for example, when I hesitate to try a new exercise, it helps me overcome my fears/doubt with the sense of confidence it provides)’. The expression in item 15 has been adapted to ‘I feel like a part of the group’. In addition, as a result of the suggestion that it would be more clear and more understandable for the Turkish society, ‘on how weightlifting technique should be, benefits of exercise etc.’ was added as an example for ‘workshop’ in item 12. The scale required approximately 10 min to complete.

### 3.2. Sample Characteristics

A total of 346 individuals were screened for this study, and 144 individuals who reported not being physically active were not allowed to participate in the survey. A total of 202 participants were included in the study. The demographic characteristics of the participants are presented in Table 1. All of the 202 participants (98 females, 104 males; mean ± SD age, 26.5 ± 6.1 years; BMI, 23.3 ± 3.2 kg/m^2^) completed the entire evaluation form at the first evaluation. Seventy participants did not complete the second evaluation. Of the 202 participants who were included in the first evaluation, 132 participants (71 females, 61 males; mean ± SD age, 26.6 ± 6.3 years; BMI, 23.2 ± 2.9 kg/m^2^) completed the second evaluation for test–retest analysis.

According to the IPAQ-SF results, 11 participants were classified as inactive, 89 participants as highly active, and 102 participants as minimally active (Table 1).

**Figure 1:**
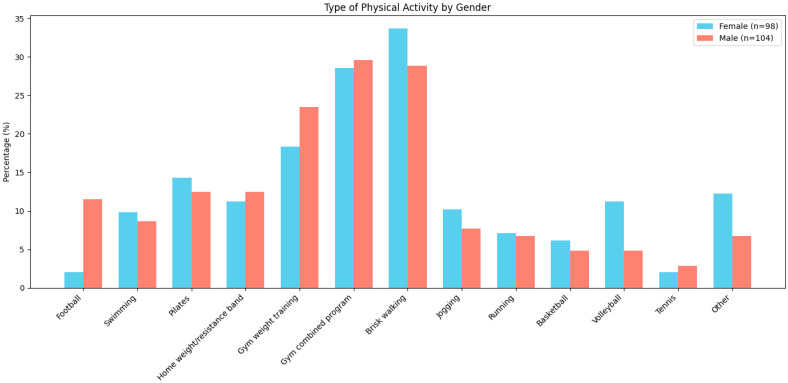
Distribution of Physical Activity Types by Gender (%).

Among female participants, the most commonly reported type of physical activity was brisk walking (33.67%), followed by combined gym programs that incorporated both resistance training and aerobic exercises (28.57%), and gym weight programs/strength training (18.36%). In male participants, the most frequently reported activities were combined gym programs (29.59%), brisk walking (28.84%), and gym weight programs/strength training (23.46%). Pilates (14.28%), swimming (9.8%), the home weight/resistance band strength training program (11.2%), volleyball (11.22%), basketball (6.12%), and other activities (12.24%)—including CrossFit, dance, spinning, Zumba, kickboxing, and rowing—were more frequently reported by female participants compared to males. In contrast, activities more commonly reported by male participants included combined gym programs (29.59%), gym weight program/strength training (23.46%), the home weight/resistance band strength training program (12.5%), football (11.53%), and tennis (2.88%) (Figure 1).

According to the descriptive statistics, the mean scores of the participants were as follows: IPAQ-SF total score: 3393.62 ± 2692.02; IPAQ-SF moderate: 640.33 ± 516.69; IPAQ-SF vigorous: 1641.58 ± 1964.67; IPAQ-SF walking: 1168.80 ± 1262.29; MSPSS: 69.17 ± 13.47; PCS-12: 52.58 ± 0.50; MCS-12: 41.61 ± 0.62; and PASSS total: 4.90 ± 0.94. The subscale scores of PASSS were as follows: emotional support: 5.40 ± 1.44, companionship support: 5.23 ± 1.49, instrumental support: 5.41 ± 1.28, informational support: 4.95 ± 1.22, and validation support: 3.54 ± 1.61 (Table 2).

**Figure 2:**
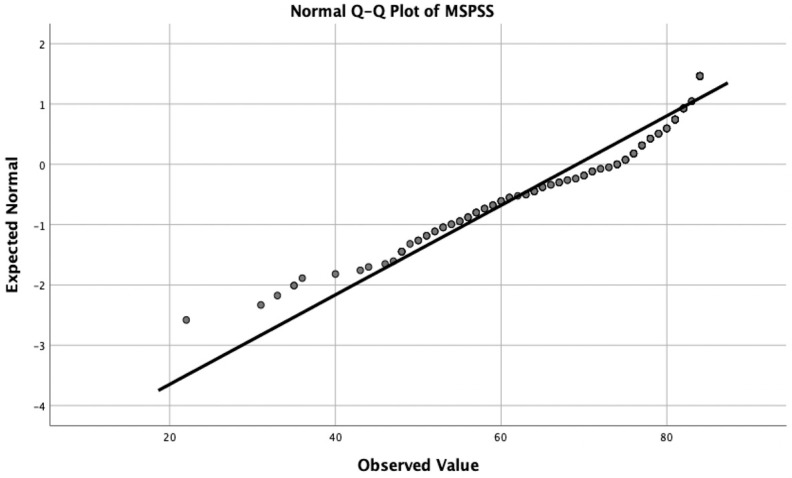
Scattergram of the MSPSS scores in the study population.

The Shapiro–Wilk test results indicated that age, IPAQ-SF, PASSS, MSPSS, PCS-12, and MCS-12 variables did not follow a normal distribution (*p* < 0.05). The scattergram of the social support level based on the MSPSS score of the study population is shown in Figure 2.

IPAQ-SF total, IPAQ-SF vigorous, and the PASSS validation scores were significantly higher in females compared to males (*p* < 0.05), whereas no significant differences were found between genders in PASSS total, sub-dimensions of PASSS except for validation, MSPSS, PCS-12, and MCS-12 (*p* > 0.05).

### 3.3. Internal Consistency

The PASSS Cronbach’s α coefficient was found to be 0.84, with good internal consistency. In the sub-dimensions of the scale, Cronbach’s α coefficients were 0.80 for emotional, 0.78 for companionship, 0.71 for instrumental, 0.71 for informational, and 0.75 for validation support.

### 3.4. Test–Retest Reliability

PASSS total ICC was found to have excellent reliability with a value of 0.90 [95% CI = 0.86–0.93]. In sub-dimensions of the scale, ICC was 0.87 [95% CI = 0.81–0.90] for emotional, 0.86 [95% CI = 0.80–0.90] for companionship, 0.88 [95% CI = 0.84–0.92] for instrumental, 0.88 [95% CI = 0.84–0.92] for informational, and 0.88 [95% CI = 0.83–0.91] for validation support.

### 3.5. Exploratory Factor Analysis (EFA)

In line with the objectives of the study, an exploratory factor analysis (EFA) was conducted to investigate the underlying factor structure of the scale. The Kaiser–Meyer–Olkin (KMO) measure of sampling adequacy was 0.796, indicating a meritorious level of sample adequacy for factor analysis. Additionally, Bartlett’s test of sphericity was statistically significant (χ^2^ = 1505.966; df = 190; *p* < 0.001), confirming that the correlation matrix was suitable for factor analysis. The EFA was performed using the maximum likelihood extraction method with Varimax rotation. Based on the criterion of eigenvalues greater than 1, a five-factor solution was identified, explaining 53.60% of the total variance. Examination of the rotated factor loadings revealed that each item loaded meaningfully on one of the five extracted factors. The five factors were interpreted and labeled based on the content of the items they comprised:

- Emotional Support (items 1–4): Represents social interactions through which participants feel emotionally supported.
- Validation Support (items 5–8): Includes feedback and comparisons that help participants assess their competence relative to others.
- Instrumental Support (items 17–20): Refers to tangible assistance facilitating participation in physical activity (e.g., equipment, childcare, transportation).
- Companionship Support (items 13–16): Reflects feelings of social belonging and being part of a group.
- Informational Support (items 9–12): Captures perceptions of receiving and sharing information related to physical activity.

An examination of communalities showed that most items exhibited moderate to high levels of shared variance, with extracted values ranging from 0.155 to 0.920. Item 2 demonstrated the highest communality, indicating strong shared variance with its corresponding factor. In contrast, items 9 and 12 had relatively low communalities, suggesting limited contribution to the overall factor structure. In addition, the chi-square goodness-of-fit test was found to be significant (χ^2^ = 189.530; df = 100; *p* < 0.001), indicating that the proposed model provides an adequate fit to the data and supports the multidimensional nature of the scale.

### 3.6. Construct Validity

The PASSS total Turkish version demonstrated fair correlation with the IPAQ-SF total (rho = 0.27, *p* < 0.05) and MSPSS (rho = 0.37, *p* < 0.05). PASSS companionship and instrumental demonstrated fair correlation with the IPAQ-SF total (rho = 0.34, rho = 0.36, respectively, *p* < 0.05) and IPAQ-SF vigorous (rho = 0.32, rho = 0.21, *p* < 0.05). PASSS emotional, companionship, instrumental, and informational demonstrated good to fair correlation with the MSPSS (rho = 0.37, rho = 0.23, rho = 0.41, and rho = 0.27, respectively, *p* < 0.05). PASSS validation demonstrated poor correlation with IPAQ-SF moderate (rho = 0.17, *p* < 0.05). Correlations between PASSS total, sub-dimensions of the PASSS, and all outcome measures are shown in Table 3.

### 3.7. Convergent and Divergent Validity

PASSS total and validation showed poor correlation with the PCS-12 (rho = 0.15, *p* < 0.05). There was no correlation between PASSS and MCS-12 (*p* > 0.05). Correlations between the sub-dimensions of PASSS and PCS-12 and MCS-12 are shown in Table 3.

### 3.8. Ceiling and Floor Effects

Only 1 participant had the lowest score (0.49%), and 4 participants had the highest score (1.98%). This is considerably below the 30% threshold that is typically used to indicate the presence of ceiling or floor effects. Therefore, it was concluded that the PASSS did not have ceiling or floor effects.

## 4. Discussion

The aim of this study was to translate, culturally adapt, and evaluate the validity and reliability of the Turkish version of the PASSS. Our results demonstrated that the PASSS has good internal consistency, sufficient validity, and excellent reliability.

The translation and cultural adaptation of the scale were carried out with the support of the scale’s developer to prevent any semantic issues arising from linguistic and cultural differences. The word “reassurance” in the original scale is mainly understood as economic support in Turkish. In order for the participants to consider this expression within the scope of emotional support, we added a sample expression that gives the meaning of the word in parentheses to the first item. Furthermore, the Turkish equivalent of the term “positive feedback” is not commonly used in everyday language; hence, item 2 has been translated as “providing positive feedback or motivating”, in order to ensure clearer understanding, considering that the purpose of positive feedback is to motivate the individual. Since the Turkish equivalent of the word “workshop” can also be understood as an activity where exercise is practiced, a clarification explaining the type of workshop has been added in parentheses in item 12 “(for example, how weightlifting technique should be, benefits of exercise, etc.)”. The following changes have been made so that people who do not have children/pets can also answer item 19: “I have someone who would watch my child(ren) or pets if needed for me to engage in the activity/activities or if I had a person or pet under my responsibility, I could find someone to watch him/her/it while I was participating in the activities”.

The study initially recruited 202 participants, with 132 completing the retest assessment. This decrease corresponds to an attrition rate of approximately 34.6%. Although some dropout occurred between assessments, previous research indicates that a sample size of at least 50 participants is sufficient for retest reliability analyses. Therefore, despite the reduction, the remaining sample size is considered adequate for reliability assessment. Nonetheless, the reasons behind participant attrition and its potential effects warrant consideration, and future studies should aim to minimize such dropout rates [27,35].

The participants’ total physical activity level (3393.62 MET-min/week) was high, comparable to the samples in the studies by Golaszewski & Bartholomew (2019) and Zou et al. (2023) (4576.5 MET-min/week and 3556.452 MET-min/week, respectively) [16,17]. Additionally, the participants’ moderate physical activity level was 640.33 MET-min/week, and their vigorous physical activity level was 1641.58 MET-min/week, and this was in accordance with the WHO’s classification of a minimum of 600 MET-minutes of moderate-intensity physical activity per week [36]. Consistent with the results of the Turkey Nutrition Health Survey (2014) [37] and studies conducted with similar age groups [38,39], the total physical activity level of males in our study was significantly higher than that of females. Although there were similar results between genders in terms of the total PASSS score, a significant difference was found in the validation sub-dimension. This may be related to the higher participation of females in group activities such as Pilates and Zumba compared to males. As stated in the literature, the social interaction in group activities may help participants feel validated and supported while exercising [22].

When examining the participants’ PASSS averages, the mean score for the validation subscore was 3.54, consistent with the original scale’s mean of 3.43 [16]; however, it was lower compared to the other subscores. The lower scores observed in the validation subscale of the PASSS indicate that participants tend to engage less in social comparison. This finding can be linked to the cultural characteristics of Turkish society, where individual performance is often evaluated within the context of social harmony and group cohesion rather than direct comparison with others. In Turkey’s collectivist culture, maintaining group unity is prioritized, which may discourage individuals from openly comparing their achievements to those of others [40]. Therefore, the lower scores in the validation subscore likely reflect a cultural tendency to avoid social comparison, emphasizing the importance of cultural context in interpreting social support. Moreover, item 8 of the validation subscore specifically addresses the comparison of performance through social media. However, the use of social media in Turkey might differ from Western countries, possibly reducing its role as a tool for social comparison [41]. Future research should further investigate these cultural factors and social comparison behaviors to improve the understanding of the PASSS’s validity across different cultures.

Golaszewski & Bartholomew (Cronbach’s α = 0.89) and Zou et al. (Cronbach’s α = 0.95) reported good and excellent values of internal consistency in original English and Chinese versions [16,17]. Similar to the original English version of the scale, the findings from this study showed that PASSS has good internal consistency (Cronbach’s α = 0.84). In our study, companionship, validation, and informational sub-dimensions showed good internal consistency (Cronbach’s α = 0.78, 0.75, and 0.71, respectively), in line with the original English version of the scale (Cronbach’s α = 0.81, 0.79, and 0.78, respectively). Emotional support was excellent (Cronbach’s α = 0.92) in the Chinese version, while it was good in the Turkish and original English versions (Cronbach’s α = 0.80 and 0.89, respectively). While the internal consistency of the instrumental sub-dimension was acceptable in the original English version (Cronbach’s α = 0.67), it was good in this study (Cronbach’s α = 0.71).

For the test–retest reliability, PASSS was performed two times. The fact that the PASSS questionnaire consisted of 7 Likert-type 20 questions reduced the recall risk. It was aimed to further reduce the probability of recall by maintaining a 7–10 day period for the test and retest interval. The test–retest reliability for the Turkish version of the PASSS with all its sub-dimensions was excellent, with an ICC score of 0.90, similar to the ICC value of 0.82 expressed as good reliability in the original English version [16].

The total score of PASSS showed fair correlations with MSPSP and poor/fair correlations with moderate, vigorous, and total activity levels on IPAQ. It has been reported in a previous study that PASSS can predict the level of physical activity [42]. There was a weak but statistically significant correlation between the PASSS and the SF-12 PCS, while no significant correlation was found between the PASSS and the SF-12 MCS. Supporting this finding, in our study, PASSS scores reflected the physical health dimension. As expected, the PASSS scores were not correlated with mental function measures, such as the SF-12 MCS. This lack of correlation was anticipated because the SF-12 MCS was specifically selected to assess divergent validity. While the PASSS measures perceived social support within the context of physical activity, the SF-12 MCS evaluates mental health-related quality of life. The weak relationship between PASSS and SF-12 MCS confirms that these scales assess distinct constructs. This supports the divergent validity of the PASSS, indicating that it captures a unique aspect of social support related to physical activity, independent from general mental health. Therefore, these results reinforce the conceptual clarity and structural validity of the PASSS and suggest that future studies should consider this distinction.

The PASSS is the first validated Turkish scale that can be used in adults to evaluate the social aspect of physical activity multidimensionally. Its adaptation to Turkish society will enable a comprehensive assessment of individuals in studies exploring the dynamic relationship between physical activity and social support. However, some limitations should be considered. First, since the data are based on participants’ self-reports, responses may be influenced by personal perceptions, inaccurate reporting, or momentary mood. Second, as the sample consists solely of young and physically active individuals, the generalizability of the findings to other age groups or individuals with lower physical activity levels is limited. Finally, collecting data online and via telephone may lead to variations in how participants interpret the questions or result in reduced attention. Future studies could further investigate the validity and reliability of the PASSS across diverse populations, including racial and ethnic minorities, individuals from different socioeconomic backgrounds, various age groups, educational levels, cultural contexts, and disadvantaged groups. Future studies could further investigate its validity and reliability across diverse populations, including racial/ethnic minorities, individuals from different socioeconomic backgrounds, various age groups, educational levels, cultural contexts, and disadvantaged groups. Additionally, examining its applicability in different sports disciplines could provide insights into the unique social support dynamics of each sport.

## 5. Conclusions

The key finding of this study was that the Turkish version of PASSS shows good internal consistency, acceptable construct validity, and excellent reliability. Therefore, the Turkish version of PASSS can be a valuable tool among the Turkish population to assess the role of social support in physical activity. In addition, this study will contribute to the validation and reliability of the PASSS scale, expand the understanding of physical activity and social support, and enhance compliance with physical activity by addressing social support mechanisms.

## Figures and Tables

**Table 1:** Demographics of the participants.

Characteristic	n = 202
Age	
Mean ± SD	26.5 ± 6.1
Range	18–53
Gender	
Male	104 (51.5%)
Female	98 (45.5%)
BMI	23.3 ± 3.2
Education	
High School	5 (2.5%)
University	159 (78.7%)
Master’s Degree	38 (18.8%)
Activity Level According to IPAQ-SF	
Inactive	11
Minimally active	102
Active	89

BMI: Body Mass Index.

**Table 2:** Descriptive Scores of The Outcome Measures.

Measure	Minimum	Maximum	Mean ± SD
IPAQ-SF total	396	20,052	3393.62 (2692.02)
IPAQ-SF moderate	0	2400	640.33 (516.69)
IPAQ-SF vigorous	0	17,280	1641.58 (1964.67)
IPAQ-SF walking	66	8316	1168.80 (1262.29)
MSPSS	22	84	69.17 (13.47)
PCS-12	23	64	52.58 (0.50)
MCS-12	15	58	41.61 (0.62)
PASSS total	1	7	4.90 (0.94)
PASSS emotional	1	7	5.40 (1.44)
PASSS companionship	1	7	5.23 (1.49)
PASSS instrumental	1	7	5.41 (1.28)
PASSS informational	1	7	4.95 (1.22)
PASSS validation	1	7	3.54 (1.61)

IPAQ-SF: International Physical Activity Questionnaire-Short Form, PASSS: Physical Activity and Social Support Scale, MSPSS: Multidimensional Scale of Perceived Social Support, PCS-12: Physical Component Score-12, MCS-12: Mental Component Score-12.

**Table 3:** Correlation Between the PASSS total, the PASSS sub-dimensions, and IPAQ-SF total, IPAQ-SF moderate, IPAQ-SF vigorous, MSPSS, PCS-12, and MCS-12, according to Spearman’s correlation coefficient.

Measure	PASSS Total (rho)	PASSS Emotional (rho)	PASSS Companionship (rho)	PASSS Instrumental (rho)	PASSS Informational (rho)	PASSS Validation (rho)
IPAQ-SF total	0.27 *	0.05	0.34 *	0.36 *	0.06	0.11
IPAQ-SF moderate	0.17 *	0.13	0.06	0.12	0.08	0.17 *
IPAQ-SF vigorous	0.22 *	0.05	0.32 *	0.21 *	0.10	0.11
MSPSS	0.37 *	0.37 *	0.23 *	0.41 *	0.27 *	0.10
PCS-12	0.15 *	0.08	0.03	0.10	0.03	0.23 *
MCS-12	0.01	0.04	0.03	0.08	0.00	0.02

IPAQ-SF: International Physical Activity Questionnaire-Short Form, PASSS: Physical Activity and Social Support Scale, MSPSS: Multidimensional Scale of Perceived Social Support, PCS-12: Physical Component Score, MCS-12: Mental Component Score, * *p* < 0.05.

## Data Availability

Data are contained within the article and Supplementary Materials.

## Supplementary Materials

The following supporting information can be downloaded at: https://www.mdpi.com/article/10.3390/healthcare13111343/s1, Physical Activity and Social Support Scale in Turkish.
