# Supplementary material for: Turkish Translation, Cross-Cultural Adaptation, Validity, and Reliability of the Physical Activity and Social Support Scale (PASSS) in Physically Active Healthy Adults
Source: Healthcare (Basel). 2025 Jun 5;13(11):1343. doi: 10.3390/healthcare13111343 (PMC12154235; doi:10.3390/healthcare13111343)
Supplement: Supplementary file 1 [file healthcare-13-01343-s001.zip › healthcare-3558096-supplementary.pdf]

Fiziksel Aktivite ve Sosyal Destek Skalası

Ankette geçen "**aktivite**" terimi, tempolu bir yürüyüş, pilates, bisiklet sürme gibi pek çok farklı egzersiz türünün yanı sıra, tırmanış, dans gibi çeşitli aktiviteleri kapsayan enerji harcanımı gerektiren ve kalp atımınızı-solunum hızınızı artıran çeşitli aktivitelerinizi tanımlar. Bir aktivite için sosyal destek, arkadaşlar, aile, canlı veya online gruplar, internet blogları, internet taramaları gibi pek çok farklı kaynaktan gelebilir. Aşağıdaki soruları cevaplarken bu kaynakları aklınızda bulundurunuz.

Lütfen aşağıda verilen ifadelerin her biri ile ilgili görüşünüzü belirtiniz (1=Kesinlikle katılmıyorum, 2=Çoğunlukla katılmıyorum, 3=Kısmen katılmıyorum, 4=Kararsızım, 5=Kısmen katılıyorum, 6= Kesinlikle katılıyorum).

1. Aktivitelerde bana duygusal destek/güvence sağlayabilecek (örneğin yeni bir egzersizi denemekten çekindiğimde sağladığı güven duygusu ile korkularımı/şüphelerimi gidermeme yardımcı olan) biri var.

2. Aktivitede/aktivitelerde beni motive eden veya olumlu geri bildirimde bulunan biri var.

3. Aktiviteyle/aktivitelerle ilgili yaşadığım sorunları veya endişelerimi anlayacak biri var.

4. Aktivitede/aktivitelerde ilişki kurabileceğim biri var.

5. Aktivitede/aktivitelerde beklentilerimi başkalarının performansına göre belirlerim.

6. Aktivitede/aktivitelerde diğer kişilerin müsabaka sonuçlarını (bir yarış/müsabaka varsa), aktiviteyi kaç defa, ne kadar sürede yaptıklarını veya ne kadar ağırlık kaldırdıklarını bilmek isterim.

7. Aktivitede/aktivitelerde kendimi diğer kişilerle karşılaştırırım.

8. Diğer insanların aktivite/aktivitelerdeki performansını kendimle karşılaştırmak için sosyal medyayı kullanırım.

9. Aktiviteye/aktivitelere ilişkin makaleler okurum.

10. Aktiviteyi/aktiviteleri daha iyi yapabilmek için başkalarına sorar, bilgi edinirim.

11. Aktiviteyle/aktivitelerle ilgili yardım istemek veya tekniğimi geliştirmek için başkaları ile konuşurum.

12. Aktiviteyi/aktiviteleri konu alan eğitimlere, workshoplara (örneğin ağırlık kaldırma tekniğinin nasıl olması gerektiği, egzersizin faydaları vb. konulu) katılırım.

13. Aktivite/aktiviteler yapan bir grubun parçasıyım.

14. Aktivite/aktiviteler yapmadığım zamanlarda, daha önce fiziksel aktivite sırasında tanıştığım insanlarla vakit geçirmeye devam ederim.

15. Kendimi birlikte aktivite yaptığım grubun bir parçası gibi hissederim.

16. Arkadaş çevremin dışında da birlikte aktivite/aktiviteler yapacak birini bulabilirim.

17. Aktivite/aktiviteleri gerçekleştirmek için ulaşım konusunda çevremden destek alabilirim.

18. Aktiviteyi/aktiviteleri yapabilmem için bana gerekli malzemeyi sağlayabilecek ya da ödünç verebilecek birileri var.

19. Aktivite / aktivitelere katılacağım zaman ihtiyaç duyarsam çocuğuma, evcil hayvanıma bakabilecek biri var. Ya da sorumluluğum altında bir kişi ya da evcil hayvan olsaydı aktivitelere katılacağımda ona/onlara bakabilecek birini bulabilirdim.

20. Aktiviteye/aktivitelere katılabilmek için bana yardımcı olacak birini kısa sürede bulabilirim.

Sosyal Desteğin Formları ile İlişkili Alt Başlıklar:
Duygusal destek: # 1-4
Bilgi desteği: # 9-12
Araçsal veya maddi destek: # 17-20
Doğrulama desteği: # 5-8
Eşlik etme veya beraberlik desteği: # 13-16
